# 

**Published:** 1915-06

**Authors:** 


CONTENTS. *Page*

Cotton-Seed Dermatitis and its Cause, Pediculoides Ventricosus. By J. A. Nixon, M.B. Cantab., F.R.C.P. ... 73

Neuritis: Its Definition and Successful Treatment. By Naunton Davies, F.R.C.S. ... 81

Women Doctors with the Army in the Field. By Helen B. Hanson, M.D., B.S.Lond. ... 88

The Clinical Examination of Stools. By G. Hely-Hutchinson Almond, M.A., B.M., B.Ch. Oxon., M.R.C.S. Eng., L.R.C.P. Lond. 95

Progress of the Medical Sciences:

Medicine ... 108

Reviews of Books ... 114

Obituary ... 123

Local Medical Notes ... 126

